# Zooplankton species composition and diversity in the seagrass habitat of Lawas, Sarawak, Malaysia

**DOI:** 10.3897/BDJ.9.e67449

**Published:** 2021-06-17

**Authors:** Johan Ismail, Abu Hena Mustafa Kamal, Mohd Hanafi Idris, S. M. Nurul Amin, Hadi Hamli, Leong Sui Sien, Abdulla Al-Asif, Muyassar H. Abualreesh

**Affiliations:** 1 Department of Animal Science and Fishery, Faculty of Agricultural Science and Forestry, Universiti Putra Malaysia Bintulu Campus, Nyabau Rd, 97008, Bintulu, Sarawak, Malaysia Department of Animal Science and Fishery, Faculty of Agricultural Science and Forestry, Universiti Putra Malaysia Bintulu Campus, Nyabau Rd, 97008 Bintulu, Sarawak Malaysia; 2 Faculty of Fisheries and Food Science, Universiti Malaysia Terengganu, 21030, Kuala Nerus, Terengganu, Malaysia Faculty of Fisheries and Food Science, Universiti Malaysia Terengganu, 21030 Kuala Nerus, Terengganu Malaysia; 3 FAO World Fisheries University, Pukyong National University, Busan, Republic of Korea FAO World Fisheries University, Pukyong National University Busan Republic of Korea; 4 Department of Marine Biology, Faculty of Marine Sciences, King Abdualaziz University, P. O. Box 80207, Jeddah 21589, Jeddah, Saudi Arabia Department of Marine Biology, Faculty of Marine Sciences, King Abdualaziz University, P. O. Box 80207, Jeddah 21589 Jeddah Saudi Arabia

**Keywords:** tropical, copepod, bivalve, mangrove, coastal, Borneo

## Abstract

Seagrass habitats are considered to be some of the most biodiverse ecosystems on the planet and safeguard some ecologically and economically important fauna, amongst which are some globally threatened species, including dugong. Malaysian seagrass ecosystems are not widespread, but their existence supports some significant marine fauna. A rigorous zooplankton study was conducted from May 2016 to February 2017, in the seagrass habitat of Lawas, Sarawak, Malaysia, to examine their temporal composition and diversity, together with their ecological influences. A total of 45 zooplankton species from 13 significant groups were recorded in the seagrass habitat. The population density of zooplankton ranged between 2,482 ind/m³ and 22,670 ind/m³ over three different seasons. A single zooplankton copepod was found to be dominant (47.40%), while bivalves were the second largest (31.8%) group in terms of total abundance. It was also noticed that the average relative abundance (0.62) and important species index (62.08) of copepods were higher than for other groups that exist in the seagrass meadow, whereas copepod *Parvocalanus crassirostris* showed both the highest average relative abundance (0.41) and the highest important species index (41.15). The diversity (*H*') and richness index of the intermediate season were found to be highest due to favourable physico-chemical conditions. Within the referred seasonal cluster, the wet and dry seasons were almost similar in terms of species abundance, while the intermediate season was distinct, with high species diversity backed by ANOSIM analysis results. Copepod and bivalves formed one group with a common similarity level of 0.80. The CCA (Canonical Correspondence Analysis) model established that abiotic factors, especially turbidity, NO_2_, rainfall, dissolved oxygen and pH were significantly correlated with abundance of individual groups of zooplankton. Zooplankton assemblage and abundance in Lawas were found to be very rich in multiple seasons, indicating that the productivity of uninterrupted seagrass habitat might be high and the system rich in biodiversity.

## Introduction

Seagrass habitats are well known for their large spectrum of ecological services, including shelter, nursing, feeding and provision of breeding places for many marine organisms, such as fishes (Ambo-Rappe et al. 2013, Du et al. 2018, Le et al. 2020, Shoji et al. 2017). These habitats serve as primary places of foraging for the different life stages of fishes (Lee et al. 2014). The biodiversity of seagrass meadows is found to be very high compared to the other marine ecosystems, with many organisms other than fishes also depending on this habitat (Duffy 2006, Hughes et al. 2009). Most planktivorous fishes gather in seagrass meadows due to the availability of plankton (Guidetti 2000). The co-existence of phytoplankton with seagrass is well documented and where abundance of phytoplankton is found to be rich, high availability of zooplankton is expected, as zooplankton is the primary consumer of primary producer phytoplankton (Alikunhi and Kathiresan 2012, Barrón et al. 2006, Setiabudi et al. 2016). However, little is known about the co-existence of zooplankton, including seagrass and the impact of zooplankton on seagrass meadows (Deepika et al. 2019, Matias-Peralta and Yusoff 2015, Melo et al. 2010, Metillo et al. 2018, Shuaib et al. 2019).

The fish feeding habit for zooplankton species varies between day and night and with presence of surface- and benthic-dwelling zooplankton species, while the presence of the maximum number of zooplankton taxa in a specific habitat, co-existing with different trophic level fishes correlates to the health of an ecosystem (Robertson and Howard 1978). Studies have shown that aquaculture activity near seagrass meadows might affect both the zooplankton community and feeding interaction of fishes and lead to disruption of prey-predator relationships and of the food web (Jaxion-Harm et al. 2013, Metillo et al. 2019). Seasonal influences cause the fluctuations in different zooplankton species, as reported from various geographical regions, for example, crustacean species in Korean seagrass beds (Park et al. 2020), seagrass meadows in the Red Sea (Abo-Taleb et al. 2020) and seagrass beds in the Gulf of Thailand (Maiphae and Sa-Ardrit 2011).

Copepods represent the major zooplankton group of primary consumers, playing a crucial role in the cycling of nutrients and energy, both in the marine ecosystem and seagrass meadows, by forming a trophodynamic link between primary (phytoplankton) and tertiary (planktivorous ﬁsh) production (De Young et al. 2004). The number of copepod species varies from place to place, with the availability of phytoplankton or other nutrients (Kassim et al. 2015, Matias-Peralta and Yusoff 2015, Shuaib et al. 2019). The number of juvenile fish individuals in seagrass nursery habitats, according to fish trophic levels, tends to be higher than in open marine waters, due to increased nutrient influx and increased productivity of phytoplankton with zooplankton (Parsons et al. 2018). As a result, the health of a seagrass ecosystem can be tracked through study of the abundance of zooplankton and together with the availability of different fish species (Ara et al. 2016).

Some zooplankton studies were performed in different habitats, including freshwater lakes, river estuaries and coastal water in both West Malaysia (Balqis et al. 2016, Johan et al. 2012, Matias-Peralta and Yusoff 2015, Rezai et al. 2011) and East Malaysia (Aiman et al. 2020, Hoque et al. 2015, Johan et al. 2013). Some authors from Malaysia have previously documented low zooplankton diversity, inclusive of copepods, within the Malaysian seagrass system, but the ecology of zooplankton and seagrass in Malaysian seagrass habitats is still relatively unknown.

Sarawak is a significant Province located in East Malaysia, where the existence of seagrass meadows is relatively confined to one place, Punang-Sari River Estuary, Lawas (Ahmad-Kamil et al. 2013, Al-Asif et al. 2020, Bujang et al. 2018, Bujang et al. 2006, Johan et al. 2020). Previous studies in this seagrass ecosystem investigated the number of species, some overall water quality parameters (Ahmad-Kamil et al. 2013), seagrass diversity (Bujang et al. 2018, Bujang et al. 2006) and macrobenthos abundance (Al-Asif et al. 2020). Nevertheless, no other study reflects the overall ecology of zooplankton together with seagrass. The present study was conducted to understand zooplankton availability, abundance and ecological interaction with seagrass meadows, to fill this knowledge gap. The findings of the present study will provide understanding of the available zooplankton community structure, their temporal distribution in seagrass meadows and associated fauna. The outcomes of this investigation are useful ultimate standards for habitat safeguarding and viable administration of the Lawas seagrass meadows, Sarawak Malaysia, South China Sea.

## Materials and Methods

### Description of the study area

**Figure 1.**
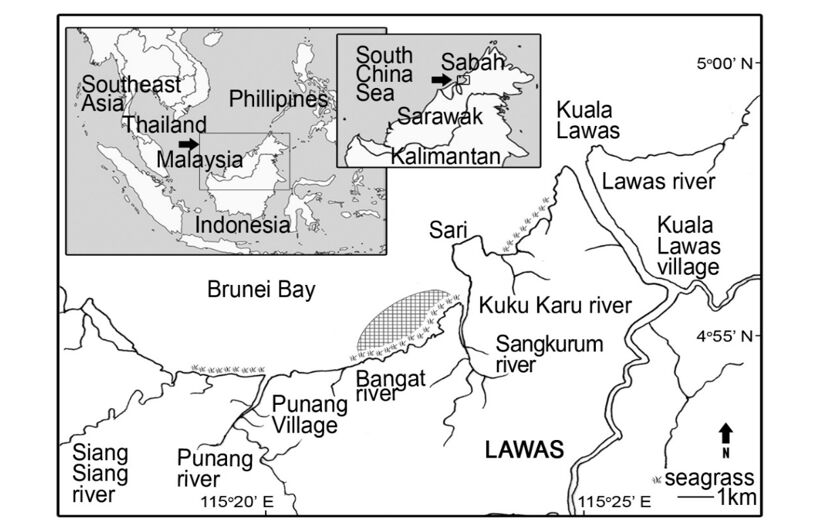
Study area showing the sampling site (shaded) at the seagrass beds in Lawas, Sarawak (adapted from Johan et al. 2020).

The seagrass habitat of Lawas is located on the south-eastern corner of the South China Sea, within Brunei Bay (Fig. 1). The seagrass bed is near to (approximately 15 km away from) the small town of Lawas (4°55'26.6"N, 115°23'30.0"E), northern Sarawak and bordering with both the State of Sabah (33 km) and Brunei (25 km). The coastal villagers in Lawas are mostly small-scale fishermen and are established in Kampung Punang, Kampung Kuala Lawas and Kampung Awat-awat. According to previous research, eight seagrass species have been recorded in the study area, namely *Halodule pinifolia*, *H. uninervis*, *Halophila ovalis*, *H. minor*, *H. beccarii*, *Cymodocea rotundata*, *Enhalus acroides* and *Thalassia hemprichii* (Ahmad-Kamil et al. 2013, Bujang et al. 2006). The seagrass habitat in Lawas co-exists with mangrove forests that are dominated by *Avicennia* sp., *Bruguiera parviflora*, *B. sexangula*, *Lumnitzera racemosa*, *L. littorea*, *Nypa fruticans*, *Rhizophora apiculata*, *R. mucronata*, *Sonneratia alba*, *S. caseolaris* and *Xylocarpus granatum* (Gandaseca et al. 2014). The study area experiences three seasonal monsoon patterns; intermediate (January till April), dry (May till August) and wet (September till December), as described by Hossain et al. (2008). The major rivers that flow into the study area are Batang Lawas, Sungai Punang, Sungai Sangkurum, Sungai Siang-Siang and Sungai Bangat.

### Collection of biological samples

Zooplankton was collected using a plankton net with a mesh size of 150 μm and diameter of 0.3 m. The plankton net was towed horizontally at a constant speed for three minutes at near-surface depth. The volume of water filtered by the plankton net was determined from a flow meter attached to the net and net dimensions. Three sampling exercises were conducted with three replications, once during each season. Three zooplankton samples were collected randomly within the study area for each season. All the samples were collected during the day time only. The zooplankton samples were preserved in 4% formalin (Omori and Ikeda 1984). The zooplankton samples were then processed for identification and counting. The total counts of zooplankton recorded from three random hauls were used to calculate the abundance of the zooplankton. Zooplankton specimens were identified according to family, genus and species levels, based on appropriate literature (Bradford-Grieve 1994, Chihara and Murano 1997, Heron and Bradford-Grieve 1995, Mulyadi 2004, Mulyadi 2003, Mulyadi 2002, Nishida 1985).

### Collection of ecological parameters

Water pH, temperature, salinity, turbidity, conductivity and dissolved oxygen were recorded *in situ* using a Hydrolab DS5X multiparameter water quality sonde. Besides, triplicate surface water samples were collected from the sampling location for further analysis. The water samples were brought to the laboratory and were tested for dissolved inorganic phosphate following the ascorbic acid method, ammonia following the Phenate method (APHA 2005), chlorophyll-a following the spectrophotometric method (Parsons et al. 1984) and nitrate following the hydrazine reduction method (Kitamura et al. 1982). Rainfall data were obtained from the Meteorological Department of Malaysia (2016-2017).

### Analysis of data

Important Species Indices (ISIs) were calculated for each taxon through the multiplication of average relative abundance and frequency data from all sampling sites, according to the methods described by Rushforth and Brock (1991). The diversity of the zooplankton community was expressed using the Shannon-Wiener Diversity Index (H′), and Shannon's Equitability Evenness Index (EH). The Margalef Richness Index and Dominance Index of zooplankton were also calculated by using PAST 4.3 software (Hammer et al. 2001, Margalef 1958, Shannon and Weaver 1964). A one-way ANOVA and Tukey test was carried out to determine the seasonal variation of the different physico-chemical parameters and ecological indices, by using SAS 9.4 software (SAS Institute. 2014). Cluster analysis was conducted by using zooplankton abundance, including each member from the copepod group, with the Bray-Curtis matrix. The total abundance of zooplankton groups was taken into consideration during the calculation of analysis of similarities (ANOSIM) by the Eucleadan method, while Canonical Correspondence Analysis (CCA) was analysed by seasonal abundance using PAST 4.3 (Hammer et al. 2001).

## Results

### Zooplankton diversity

A total of 45 zooplankton species were identified and documented from the seagrass bed of Lawas, which belonged to 13 significant groups of zooplankton comprising copepods, cnidarians, bivalves, gastropoda, cladocerans, lucifer, mysids, chaetognaths, appendicularian, larvae of polychaeta, larvae of crustacean, larvae of echinoderm and fish larvae (Table 1).

### Seasonal abundance of zooplankton

The population density of zooplankton ranged from 2,482.3 ind/m³ to 22,670.0 ind/m³ in three different seasons. The single dominant group copepod had the highest abundance in the intermediate season (8,827.33 ± 3,228.95 ind/m³), followed by the wet season (3,491.00 ± 1,252.38 ind/m³) and dry season (1,610.67 ± 1,095.29 ind/m³), respectively. Larvae of bivalves was the other major group found besides copepods, with the highest abundance of bivalve observed in the intermediate season (8,787.67 ± 1,711.78 ind/m³), followed by the dry season (445.0 ± 298.82 ind/m³) and wet season (111.33 ± 45.32 ind/m³) (Table 2).

The major groups of zooplankton during the study periods were non-copepod (52.6%), while the copepod group comprised 47.40%, whereas a single copepod was found dominant. Non-copepods included: larvae of Bivalvia (31.80%), larvae of Gastropoda (11.8%), larvae of Crustacean (2.36%), Chaetognatha (2.22%), larvae of Polychaeta (1.90%), Appendicularia (1.73%) and others (0.78%) inclusive of Cladocera (0.25%), larvae of Cnidaria (0.17%), larvae of Mysida (0.12%), larvae of Echinoderm (0.02%), larvae/egg of fishes (0.06%), Luciferidae (0.08%) and larvae of Actinotroch (0.08%). The intermediate season showed an abundance of copepod (38.94%) and bivalves (38.76%) that was almost similar, but, in the wet and dry season, copepod was the largest group in terms of zooplankton abundance, at 82.46% and 64.89%, respectively.

### Zooplankton assemblage

The average abundance within zooplankton groups revealed that copepod (13,929.3 ± 2,161.47 ind/m³) (47.4%) was the most abundant in all three seasons, amongst all groups, followed by larvae of Bivalvia (9,344.24 ± 2838.11 ind/m³), larvae of Gastropoda (3,468.92 ± 1067.90 ind/m³), larvae of Crustacean (693.67 ± 91.03 ind/m³) and larvae of Echinoderm (4.80 ± 1.60 ind/m³) with the lowest abundance (Table 3). The average relative abundance maintained a similar trend to the average abundance, where copepod (0.62) was the highest in relative abundance, followed by larvae of Bivalvia (0.20), larvae of Gastropoda (0.07), larvae of Crustacean (0.04) and larvae of fishes (0.0007) with the lowest relative abundance. The frequency of most species was almost 100% in every season, as members from most of the groups were observed in every season. The important species index showed that copepod (62.08) was the most important zooplankton group in the seagrass habitat, whereas Bivalvia (19.76) and Gastropoda (7.02) also had importance to maintain the biotic integrity of the seagrass habitat (Table 3).

As copepod is a significant and abundant zooplankton group found at Lawas seagrass habitat, the present study also focused on species composition, average relative abundance, frequency and important species index of this group. Where revealed, the Relative Abundance (RA) and Important Species Index (ISI) of *Parvocalanus crassirostris* (RA; 0.41 and ISI; 41.15) was the highest amongst all copepod species and found in every season of the year (frequency, 100), followed by *Bestiolina similis* (RA; 0.13 and ISI; 12.82), *Oithona simplex* (RA; 0.12 and ISI; 11.52), *Pontellidae* sp.1 (RA; 0.1and ISI; 9.51) and so on (Table 4).

### Ecological indices

**Figure 2.**
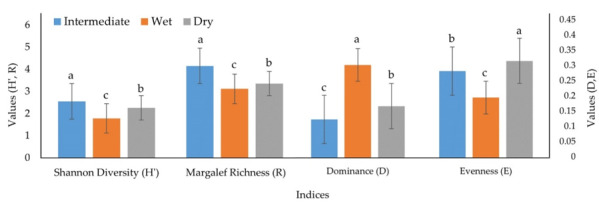
Ecological indices of zooplankton in the seagrass bed of Lawas (mean ± SE).

The intermediate season recorded the highest number of species (45 species/group) or groups of zooplankton amongst all seasons, followed by the wet and dry seasons (both with 30 species/group). The wet season (0.31) showed a significantly (*p* < 0.0001) higher Simpson Dominance Index, followed by the dry season (0.17) and intermediate season (0.13). The Diversity Index was significantly (*p* < 0.0001) higher in the intermediate season (2.55), followed by the dry season (2.26) and wet seasons (1.78), while the Evenness Index was found significantly (*p* < 0.0001) highest at dry season (0.32). Species Richness Index was found significantly (*p* < 0.0001) higher in the intermediate season (4.15), followed by dry (3.34) and wet seasons (3.11) (Fig. 2; Different superscripts within the same index indicates significant differences (*p* < 0.05)).

### Ecological parameters

Amongst all the parameters, dissolved oxygen was found significantly different (*p* < 0.03) in all three seasons, where dissolved oxygen in the intermediate season was found the highest (6.66 mg/l) and lowest in the dry season (3.76 mg/l). Water-NH_4_ concentration was found significantly different (*p* < 0.0005) in all three seasons, where the dry season showed the highest (0.52 mg/l) NH_4_ concentration and intermediate season the lowest (0.08 mg/l). Water-NO_2_ concentration was found significantly different (*p* < 0.0001) in all three seasons, where the intermediate season showed the highest (0.39 mg/l) and the dry season the lowest (0.04 mg/l). Rainfall was found significantly different (*p* < 0.0001) in all three seasons, where the intermediate season showed the highest rainfall (706.10 mm) and dry season the lowest (515.75 mm) (Table 5). The data of all ecological parameters were adopted from Johan et al. (2020).

### Cluster analysis

**Figure 3.**
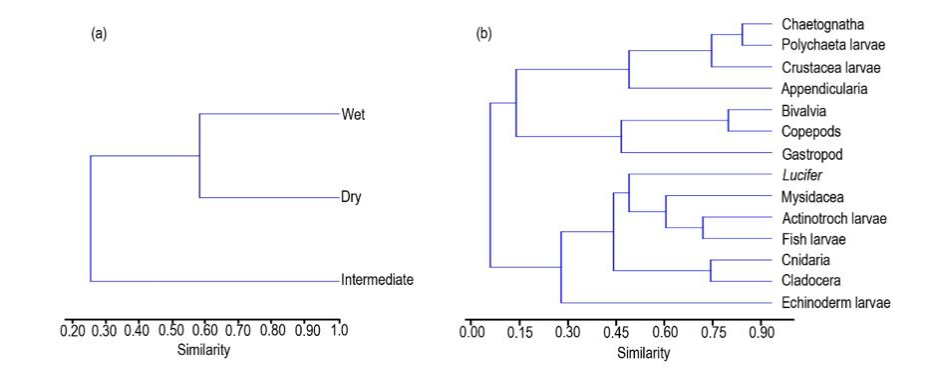
Dendrogram of cluster analysis using Bray-Curtis similarity distance, based on zooplankton density in Lawas seagrass area for different (a) seasons and (b) zooplankton groups.

Cluster analysis of zooplankton abundance, based on Bray-Curtis, showed a clear inter-seasonal grouping in all three seasons. The dendrogram presents zooplankton density in three seasons, generally classified into two groups at the similarity level of 0.58, based on the difference of seasons (Cophen. Correlation, 0.9624) (Fig. 3).

The intermediate season is separated from dry and wet seasonal clusters, which indicates that the intermediate season was found very different from the other two seasons. Cluster analysis of zooplankton abundance in species and groups, based on Bray-Curtis (Cophen. Correlation, 0.9387), showed several similar groups, where copepods and bivalves together formed one group with a similarity level of 0.80, indicating that these two groups of zooplankton had the highest abundance in all three seasons (Fig. 3)

### ANOSIM analysis

The ANOSIM analysis revealed that the dry and wet seasons had very similar species abundance, with the intermediate season found to be very dissimilar to wet (similarity index, 0.1075) and dry seasons (similarity index, 0.0966). However, the wet season was found to be very similar to the dry season (similarity index, 0.5943).

### Canonical Correspondence Analysis (CCA)

**Figure 4.**
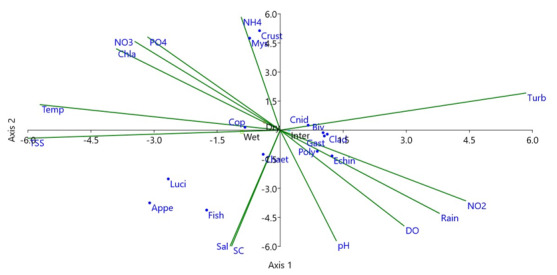
Canonical Correspondence Analysis (CCA) ordination showed the relationship between zooplankton abundance and abiotic variables.

The first Canonical axis of the variance in zooplankton abundance accounted for 90.81% (Eigenvalue, 0.14) and the second axis accounted for 9.19% (Eigenvalue, 0.01). Thus, the first two axes comprised cumulative 100% of the variance. The CCA model confirmed that key abiotic factors, turbidity, NO_2_, rainfall, dissolved oxygen and pH, were all highly correlated with the individual group of zooplankton abundance; where turbidity (Eigenvalue, 0.97), NO_2_ (Eigenvalue, 0.73), total rainfall (Eigenvalue, 0.66), dissolved oxygen (Eigenvalue, 0.49) and pH (0.22) were positively correlated to zooplankton abundance in the first axis, while salinity (Eigenvalue, -0.99) and specific conductivity (Eigenvalue, -0.99) both showed negative correlation with zooplankton abundance in the second axis (Fig. 4).

## Discussion

The present study exhibits the distribution, seasonal zooplankton dynamics and ecological abiotic factors that impact the zooplankton population in Malaysia's tropical seagrass habitat. Previous studies have denoted planktonic communities as indicators of water quality (Li and Chen 2020, Webber et al. 2005). The health of closed, open and marine water bodies can also be predicted and determined by the presence of some planktonic groups (Abdullah Al et al. 2018, Ismail and Adnan 2016, Parmar et al. 2016). As the seagrass ecosystem is very rich in biodiversity and acts as a habitat for many fishes, the importance of zooplankton presence in seagrass meadows was assessed. A total of 45 species or groups of zooplankton, from 13 prominent families or (sub-) groups, was recorded from the seagrass habitat of Punang-Sari River Estuary, Lawas, a number which is lower than the number of species (65 sp.) recorded by Deepika et al. (2019) within the seagrass ecosystem of Mandapam coast in Gulf of Mannar, India. Researchers have reported demersal zooplankton communities in mangrove (88 sp.) (Melo et al. 2010) and salt marsh estuary (33 sp.) (Abu Hena et al. 2016). Matias-Peralta and Yusoff (2015) found 48 species of zooplankton in the Merambong Seagrass Meadow and the Tinggi and Sibu Islands, Malaysia (129 sp.) (Metillo et al. 2018).

The present study revealed the zooplankton ranges from 2,482.33 ind/m³ to 22,670.0 ind/m³ in the three mentioned seasons, where the intermediate season (22,670 ± 6,198.62 ind/m³) recorded the highest zooplankton abundance amongst seasons. Comparative zooplankton studies with zooplankton number and abundance are recorded in Table 6. The zooplankton recorded in Indian seagrass meadows (89,300 to 935,300 ind/m^3^) by Deepika et al. (2019) was far higher than densities recorded in the present study. In contrast, Melo et al. (2010) found far lower zooplankton abundance (4,759 to 7,113 ind/m^3^) in the south-western Atlantic than the present study and the zooplankton abundance (3,030.1 ± 855.6 ind/m³) at Merambong shoal seagrass area, from the findings of Azmi et al. (2016), are also lower than densities recorded in the present study. Study of some river estuaries from the Sarawak Region (Malaysia) has recorded zooplankton density ranges between 447.5 and 27,812.9 ind/m³ (Aiman et al. 2020).

Studies have revealed that, as a single group, copepod comprises a significant portion of zooplankton in different habitats, including estuarine, mangrove and seagrass (Abu Hena et al. 2016, Matias-Peralta and Yusoff 2015, Shuaib et al. 2019). In the present study, copepods occupied 47.4% of total recorded species, where as a single group, copepods were the highest in percentage. The total non-copepod (52.6%) group occupied a higher percentage than copepods. Larvae of bivalves were recorded as the second largest zooplankton group (31.8%) in the study area. Matias-Peralta and Yusoff (2015) analysed 51.2% of copepods amongst all zooplankton in Merambong Seagrass Meadow, Johor, Peninsular Malaysia, which is an area relatively similar to the present study area. Both studies present similar results and it is anticipated that various physical factors, such as sampling gear, period and area of exposure could explain dissimilar outcomes concerning species composition (Johan et al. 2013).

Copepod, ranged from 1,610.67 ± 1,095.29 to 8,827.33 ± 3,228.95 ind/m³ in dry and intermediate seasons, with an average of 13,929.3 ± 2,161.47 ind/m³, followed by larvae of Bivalvia 31.80%, larvae of Gastropoda 11.8%, larvae of Crustacean 2.36%, Chaetognatha 2.22%, larvae of Polychaeta 1.90%, Appendicularia 1.73% and others (0.78%). The intermediate season being found rich in various species and groups might have been due to nutritional abundance, availability of rich phytoplankton and ocean current. However, the present findings are similar to the studies of Deepika et al. (2019), Azmi et al. (2016), Melo et al. (2010), Matias-Peralta and Yusoff (2015) and Aiman et al. (2020).

The relative abundance of zooplankton followed the abundance pattern, such that copepods (0.62) were the highest in average relative abundance, followed by larvae of Bivalvia (0.20), larvae of Gastropoda (0.07), larvae of Crustacean (0.04) and fish larvae (0.0007) with the lowest relative abundance. Abdul et al. (2016) revealed a relative abundance of rotifer that was higher than any zooplankton species, but this study was conducted in an estuary, while the present study was conducted in a different habitat. Melo et al. (2010) revealed that the relative abundance of copepods was always higher than any other zooplankton groups. The Important Species Index showed that copepod (62.08) was the most important zooplankton group within the seagrass habitat, in all seasons, where Bivalvia (19.76) and Gastropoda (7.02) also had importance in maintaining the biotic integrity of the seagrass habitat.

As copepods were the largest zooplankton group, the current study has accounted for copepod zooplankton as the most important biotic fauna in seagrass meadows. We have calculated the relative abundance (RA) and Important Species Index (ISI) of all available copepods in Lawas. *Parvocalanus crassirostris* was the highest in density amongst all copepod species and found in every season of the year, followed by *Bestiolina similis*, *Oithona simplex*, *Pontellidae* sp.1, *Dioithona oculata*, *Acartia* sp., *Temora turbinata*, *Paracalanus parvus parvus*, *Acartia erythraea*, *Ditrichocorycaeus andrewsi*, *Oithona fallax*, *Parvocalanus elegans* and so on. Melo et al. (2010) discussed the species-specific average relative abundance of copepods in similar discussion within a prior study, but the Important Species Index has not been previously applied to the study of zooplankton in Malaysia. Ahmad et al. (2011) introduced the Important Species Index in the study of benthos in Teluk Aling, Pulau Pinang, Malaysia, where they showed the Important Species Index of gastropod *Umbonium vestiarum* was the highest amongst investigated species because that gastropod was most abundant in that study area.

The majority of the copepod species from the genus of *Paracalanus*, *Oithona* and *Acartia* are predominant in Malaysian waters and especially abundant in the nearshore and within estuaries (Chew and Chong 2011). The copepod species *P. crassirostris*, *P. parvus* and *Bestiolina similis* are established dominant species in the coastal waters of Malaysia (Johan et al. 2013, Matias-Peralta and Yusoff 2015, Rezai et al. 2004). The copepod species, *P. crassirostris* was also reported to be dominant in estuarine waters (Alvarez-Silva and Gómez-Aguirre 1994, Mazzocchi and d’Alcalà 1995). *Oithona simplex* was reported to dominate inshore and shallow waters and to be suited to low salinity water, as well as being abundant in mangrove estuaries (Johan et al. 2013). The copepod species, mentioned above, are grazing copepods, feeding mainly on detritus and phytoplankton, thus their distribution and abundance are closely related to food availability (Chew et al. 2012). The lower relative abundance of species affiliated more to oceanic origins, such as *Microsetella*, *Acrocalanus*, *Tortanus*, *Corycaeus*, *Canthocalanus* and *Temora* indicate that estuarine-dominant copepod species have more influence over the structure of the estuarine copepod community.

Fluctuations in zooplankton communities and their distribution have noteworthy impacts on fishery resources because of the significant role they play within the aquatic food web. The temporal changes in abundance of zooplankton affect the availability of dependent species, fishes for example. Temporal variation of zooplankton in the current study of Lawas seagrass meadows, revealed that the abundance of bivalves (38.76%) and copepods (38.94%) were almost similar in the intermediate season, but in the wet and dry seasons, copepods formed the largest, most abundant zooplankton group at 82.46% and 64.89%, respectively. Shi et al. (2020) provide data that support spring as the season of the highest zooplankton abundance (9,435.8 to 16,746.9 ind/m^3^) in the Yellow Sea, China, wherein copepod was still the largest group. Another study by Magalhães et al. (2009) in a tropical Amazon Estuary, northern Brazil, found zooplankton abundance was comparatively higher in the wet season and copepod was reported as the largest group. Aiman et al. (2020) presented data for April and December as the highest zooplankton abundant periods in Malaysian estuaries. Pitchaikani and Lipton (2015) showed that seasonal patterns, influenced by the prevailing monsoonal system on the east coast of India, directly influenced the presence of zooplankton. Another study by Giering et al. (2019) established a relationship between zooplankton abundance and season. Seasonal variation of zooplankton abundance in seagrass meadows was established by Deepika et al. (2019) and Matias-Peralta and Yusoff (2015).

In the present study, the Diversity Index was the highest in the intermediate season (2.55), followed by the dry season (2.26) and the wet season (1.78), while the Evenness Index was found the highest in the dry season (0.32). Species Richness Index was found the highest in the intermediate season (4.15), followed by the dry (3.36) and wet seasons (3.11), which it was found similar to other studies, including those of Aiman et al. (2020), Abu Hena et al. (2016), Ismail and Zaidin (2015) and Deepika et al. (2019).

Water quality plays a vital role to maintain zooplankton abundance, with some parameters considered significant, such as dissolved oxygen. In the present study, dissolved oxygen was the highest in the intermediate season and lowest in the dry season. Water-NH_4_ concentration was found significantly different (*p* < 0.0005) in all three seasons, with the dry season showing the highest NH_4_ concentration and intermediate season the lowest. The NO_2_ concentration was found significantly different (*p* < 0.0001) in all three seasons, with the intermediate season showing the highest concentration and dry season the lowest. Rainfall was found significantly different (*p* < 0.0001) in all three seasons with the intermediate season having the highest, and dry season the lowest rainfall. The present findings are very similar to the studies of Aiman et al. (2020), Deepika et al. (2019) and Abu Hena et al. (2016).

Cluster analysis of zooplankton abundance, based on Bray-Curtis, showed a clear inter-seasonal and inter-group clustering in all three seasons. Two clear groups were formed in seasonal clustering at the similarity level of 0.58, but as several group clusters. Amongst these, the most crucial cluster was the bivalve-copepod group, which was the most abundant group in all seasons with a similarity level of 0.80. A similar cluster analysis was performed by Aiman et al. (2020) and Johan et al. (2012) in Malaysia and Melo et al. (2010) in the south-western Atlantic.

Canonical Correspondence Analysis (CCA) revealed some key abiotic factors, including turbidity, NO_2_ concentration, rainfall, dissolved oxygen and pH, which were highly correlated with an individual group of zooplankton abundance. Aiman et al. (2020), Abu Hena et al. (2016) and Metillo et al. (2018) have provided similar types of CCA, elsewhere.

## Conclusions

The seagrass meadows of Punang-Sari Estuary, Lawas, are very rich in species diversity, including zooplankton, fishes and macrobenthos, which contribute ecologically and economically to both the alpha biodiversity and the local population, respectively. Abundance of year-round zooplankton will ensure the availability of a variety of fishes and support some ecologically and economically essential species within the area. As seagrass meadows are such a productive habitat, made rich by the presence of zooplankton, zooplankton can be considered for establishment as a baseline indicator in this habitat. Further study of zooplankton abundance, composition and ecology on available fish species is recommended.

## Figures and Tables

**Table 1.** Major groups of zooplankton recorded at seagrass bed of Lawas.

Taxa	
**Phylum**Arthropoda **Class**Hexanauplia **Order**Calanoida *Acartia erythraea* *Acartia pacifica* *Acartia* sp. *Canthocalanus pauper* *Centropages furcatus* *Acrocalanus gibber* *Acrocalanus gracilis* *Bestiolina similis* *Delibus nudus* *Parvocalanus crassirostris* *Parvocalanus elegans* *Paracalanus parvus parvus* *Calanopia* sp. *Labidocera pavo* Pontellidae *Pseudodiaptomus* sp. *Temora turbinata* *Tortanus barbatus* **Order**Cyclopoida *Oithona attenuata* *Oithona fallax* *Dioithona oculata* *Oithona plumifera* *Oithona simplex* *Hemicyclops* sp. *Ditrichocorycaeus andrewsi* *Ditrichocorycaeus asiaticus* *Ditrichocorycaeus erythraeus* *Ditrichocorycaeus subtilis* *Oncaea* sp.	**Phylum**Arthropoda **Class**Hexanauplia **Order**Mormonilloida Mormonillidae **Order**Harpacticoida *Clytemnestra scutellata* *Microsetella norvegica* *Euterpina acutifrons* *Nitokra* sp. **Class**Branchiopoda Cladocera **Class**Malacostraca Mysid **Class**Decapoda *Lucifer* sp. Crustacea larvae **Phylum**Chaetognatha *Sagitta* sp. **Phylum**Mollusca **Class**Bivalvia Bivalvia larvae **Class**Gastropoda Gastropoda larvae **Phylum**Polychaeta Polychaeta larvae **Phylum**Cnidaria Cnidaria larvae **Phylum**Echinodermata Echinodermata larvae **Phylum**Phoronida Phoronida larvae **Phylum**Chordata Class Appendicularia *Oikopleura* sp.

**Table 2.** Seasonal zooplankton distribution (mean value ± SE, ind/m^3^) of seagrass beds in Lawas Estuary.

**Zooplankton Group**	**Intermediate**	**Wet**	**Dry**
**Copepod**	8827.33 ± 3228.95^a^ (38.94%)	3491.00 ± 1252.38^a^ (82.46%)	1610.67 ± 1095.29^a^ (64.89%)
** Cnidaria **	43.33 ± 43.33^a^ (0.19%)	2.67 ± 2.67^a^ (0.06%)	3.67 ± 2.33^a^ (0.15%)
** Bivalvia **	8787.67 ± 1711.78^a^ (38.76%)	111.33 ± 45.32^b^ (2.63%)	445.0 ± 298.82^b^ (17.93%)
** Gastropoda **	3291.33 ± 608.68^a^ (14.52%)	36.0 ± 17.09^b^ (0.85%)	142.0 ± 34.95^b^ (5.72%)
** Cladocera **	71.67 ± 57.32^a^ (0.32%)	0.00 ± 0.00^a^ (0%)	3.0 ± 3.0^a^ (0.12%)
** Luciferidae **	9.67 ± 9.67^a^ (0.04%)	13.0 ± 8.14^a^ (0.31%)	2.0 ± 1.0^a^ (0.08%)
** Mysida **	20.0 ± 10.02^a^ (0.09%)	6.33 ± 3.28^a^ (0.15%)	9.67 ± 6.89^a^ (0.39%)
** Chaetognatha **	477.67 ± 251.42^a^ (2.11%)	139.33 ± 92.96^a^ (3.29%)	36.0 ± 31.51^a^ (1.45%)
** Appendicularia **	174.67 ± 36.04^a^ (0.77%)	305.0 ± 204.78^a^ (7.20%)	28.33 ± 23.85^a^ (1.14%)
**Polychaeta larv.**	522.67 ± 62.52^a^ (2.31%)	24.0 ± 16.65^b^ (0.57%)	10.67 ± 6.69^b^ (0.43%)
**Crustacean larv.**	405.00 ± 156.56^a^ (1.79%)	97.33 ± 37.74^a^ (2.30%)	191.33 ± 37.34^a^ (7.71%)
**Echinoderm larv.**	4.67 ± 4.67^a^ (0.02%)	0.00 ± 0.00^a^ (0%)	0.00 ± 0.00^a^ (0%)
**Actinotroch larv.**	24.67 ± 12.81^a^ (0.11%)	0.00 ± 0.00^a^ (0%)	0.00 ± 0.00^a^ (0%)
**Fish larvae**	9.67 ± 4.84^a^ (0.04%)	7.33 ± 4.06^a^ (0.17%)	0.00 ± 0.00^a^ (0%)
**Total**	22670 ± 6198.62	4233.33 ± 1685.073	2482.33 ± 1541.67

**Different superscripts within the same row indicate significant differences (*p* < 0.05).

**Table 3.** Zooplankton groups with their total abundance (mean value ± SE, ind/m^3^), mean Relative Abundance (RA), occurrence frequency (%F) and Important Species Index (ISI).

**Zooplankton Group**	**Total Abundance**	**RA**	**F**	**ISI**
**Copepod**	13929.3 ± 2161.47 (47.4%)	0.62	100.0	62.08
** Cnidaria **	49.67 ± 13.39 (0.17%)	< 0.01	100.0	0.13
** Bivalvia **	9344.24 ± 2838.11 (31.8%)	0.20	100.0	19.76
** Gastropoda **	3468.92 ± 1067.90 (11.8%)	0.07	100.0	7.02
** Cladocera **	74.53 ± 23.30 (0.25%)	< 0.01	66.7	0.10
** Luciferidae **	24.63 ± 3.22 (0.08%)	< 0.01	100.0	0.14
** Mysida **	35.86 ± 4.07 (0.12%)	< 0.01	100.0	0.21
** Chaetognatha **	653.34 ± 133.44 (2.22%)	0.02	100.0	2.28
** Appendicularia **	508.07 ± 79.87 (1.73%)	0.03	100.0	3.04
**Polychaeta larv.**	557.34 ± 168.55 (1.9%)	0.01	100.0	1.10
**Crustacean larv.**	693.67 ± 91.03 (2.36%)	0.04	100.0	3.93
**Echinoderm larv.**	4.80 ± 1.60 (0.02%)	< 0.01	33.3	< 0.01
**Actinotroch larv.**	24.67 ± 8. 22 (0.08%)	< 0.01	33.3	0.01
**Fish larvae**	17.0 ± 2.91 (0.06%)	< 0.01	66.7	0.05

**Table 4.** Copepod species with their annual mean Relative Abundance (RA) and Important Species Index (ISI).

Species	RA	ISI	Species	RA	ISI
*Acartia erythraea*	0.02	1.51	*Labidocera pavo*	0.0007	0.03
*Acartia pacifica*	0.003	0.13	*Microsetella norvegica*	0.0003	0.01
*Acartia* sp.	0.04	4.02	Mormonillidae sp.	0.004	0.42
*Acrocalanus gibber*	0.003	0.32	*Nitokra* sp.	0.005	0.54
*Acrocalanus gracilis*	0.0003	0.01	*Oithona attenuate*	0.0007	0.05
*Bestiolina similis*	0.13	12.82	*Oithona fallax*	0.02	1.23
*Calanopia* sp.	0.0001	0.006	*Dioithona oculate*	0.04	4.04
*Canthocalanus pauper*	0.004	0.41	*Oithona plumifera*	0.001	0.06
*Centropages furcatus*	0.004	0.27	*Oithona simplex*	0.12	11.52
*Clytemnestra scutellata*	0.0005	0.02	*Oncaea* sp.	0.0008	0.03
*Ditrichocorycaeus andrewsi*	0.01	1.39	*Parvocalanus crassirostris*	0.41	41.15
*Ditrichocorycaeus asiaticus*	0.0001	0.006	*Parvocalanus elegans*	0.02	1.18
*Ditrichocorycaeus erythraeus*	0.0007	0.02	*Paracalanus parvus parvus*	0.02	1.6
*Ditrichocorycaeus subtilis*	0.006	0.67	Pontellidae sp.	0.1	9.51
*Delibus nudus*	0.009	0.65	*Pseudodiaptomus* sp.	0.0003	0.01
*Euterpina acutifrons*	0.008	0.84	*Temora turbinate*	0.02	1.72
*Hemicyclops* sp.	0.005	0.5	*Tortanus barbatus*	0.001	0.1

**Table 5.** Summary result of two-way ANOVA and Tukey HSD test on various abiotic factors.

**Water quality parameters**	**Intermediate**	**Wet**	**Dry**	***p*-value**
**Temperature (⁰C)**	27.03 ± 0.14^a^	29.79 ± 0.29^a^	29.26 ± 1.47^a^	> 0.05
**pH**	7.88 ± 0.04^a^	7.72 ± 0.02^a^	7.10 ± 0.45^a^	> 0.05
**Salinity (PSU)**	25.63 ± 0.04^a^	27.31 ± 0.51^a^	20.20 ± 4.72^a^	> 0.05
**Conductivity (mS/cm)**	40.10 ± 0.05^a^	42.47 ± 0.73^a^	32.19 ± 6.75^a^	> 0.05
**DO (mg/l)**	6.66 ± 0.03^a^	5.31 ± 0.05^ab^	3.76 ± 0.93^b^	< 0.05
**Turbidity (NTU)**	52.90 ± 28.29^a^	28.83 ± 1.48^a^	45.17 ± 8.41^a^	>0.05
**NH_4_ (mg/l)**	0.08 ± 0.01^b^	0.14 ± 0.07^b^	0.52 ± 0.01^a^	< 0.05
**NO_3_ (mg/l)**	0.63 ± 0.17^a^	0.84 ± 0.23^a^	1.01 ± 0.22^a^	> 0.05
**NO_2_ (mg/l)**	0.39 ± 0.02^a^	0.14 ± 0.02^b^	0.04 ± 0.02^c^	< 0.0001
**PO_4_ (mg/l)**	0.002 ± 0.00^a^	0.006 ± 0.00^a^	0.02 ± 0.02^a^	> 0.05
**TSS (mg/l)**	15.64 ± 1.79^a^	41.40 ± 9.99^a^	29.67 ± 6.68^a^	> 0.05
**Chl *a* (mg/m^3^)**	0.10 ± 0.02^a^	0.84 ± 0.43^a^	1.27 ± 0.53^a^	> 0.05
**Rainfall (mm)**	706.10 ± 0.00^a^	589.38 ± 0.00^b^	515.75 ± 0.00^c^	< 0.0001

Values mean ± SE; **Different superscripts within the same row indicate significant differences (*p* < 0.05) (Adopted from Johan et al. 2020)

**Table 6.** Comparison of zooplankton abundance with other studies in the different habitat.

**Habitat**	**Abundance (ind/m^3^)**	**Mesh size**	**Reference**
Seagrass meadow Johor, Malaysia	17.0 to 104.00	100 μm	Matias-Peralta and Yusoff (2015)
Seagrass bed, Merambong shoal	3,030.16 to 4,006.50	140 μm	Azmi et al. (2016)
Seagrass bed, Pulau Tinggi, Johor	1,245.00	100 μm	Shuaib et al. (2019)
Lupar & Sadong river estuary, Sarawak	447.50 to 27812.90	150 μm	Aiman et al. (2020)
Bintulu coastal water, Sarawak	183 to 7,238.00	153 μm	Johan et al. (2013)
Seagrass bed, south-western Atlantic	7,113.00	300 μm	Melo et al. (2010)
Seagrass bed, Mandapam Coast	935,300.00	NA	Deepika et al. (2019)
Seagrass, Punang-Sari Estuary, Lawas	2482.33 to 22670.00	150 μm	Present study

## Hosting institution

Universiti Putra Malaysia
